# Feasibility trial of an integrated treatment “Activate for Life” for physical and mental well-being in older adults

**DOI:** 10.1186/s40814-022-01000-8

**Published:** 2022-02-11

**Authors:** Melba A. Hernandez-Tejada, Alexis Nagel, Mohan Madisetti, Sundar Balasubramanian, Teresa Kelechi

**Affiliations:** 1 Department of Psychiatry and Behavioral Sciences, McGovern Medical School, The University of Texas Health Science Center, Houston, TX USA; 2 College of Nursing, Medical University of South Carolina, Charleston, SC USA; 3 Department of Radiation Oncology, College of Medicine, Medical University of SC, Charleston, SC USA

**Keywords:** Feasibility, Patient satisfaction, Balance, Physical strength, Yoga, Mental health, Behavioral activation, Older adults

## Abstract

**Background:**

Pain and fatigue are common chronic conditions faced by older adults. Integrated interventions to address pain and fatigue may therefore be particularly useful for older adults, especially those interventions that target mobility and psychosocial well-being. The present study describes feasibility and participant satisfaction for an integrated eHealth treatment to address pain and fatigue in a sample of older adults living in a low-income independent residence facility and their own homes in the community.

**Methods:**

Three treatment combinations were compared in a randomized repeated measures design to determine if adding components of breathing retraining and behavioral activation to the existing Otago program (for strength and balance) affected feasibility and patient satisfaction. Specifically, 30 older adults were randomly allocated to: Arm1: the Otago alone (*n* = 10); Arm 2: Otago + Gentle Yoga and Yogic Breathing (*n* = 10); or Arm 3: Otago + Gentle Yoga and Yogic Breathing + Behavioral Activation (combination was named ‘Activate for Life’ *n* = 10). Feasibility measures included recruitment rate, session completion characteristics, and satisfaction with the program.

**Conclusion:**

Data from this study provide support for the feasibility of an integrated program to address physical and mental well-being of older adults. Future fully powered studies should now focus on assessment of clinical outcomes and refinement of individual components.

**Trial registration:**

Registered in clinicaltrials.gov with the identifier: NCT03853148.

**Supplementary Information:**

The online version contains supplementary material available at 10.1186/s40814-022-01000-8.

## Key messages regarding feasibility

- What uncertainties existed regarding the feasibility?
  Prior to conducting this pilot study, it remained unclear whether combining three different components to address functional, and mental health outcomes in low-income older adults living independently in the community was possible.
- What are the key feasibility findings?
  That participants will agree, and will engage in such an integrated treatment. Additionally, findings related to satisfaction with the integrated treatment are key, as are reports that participants continued activities post-treatment.
- What are the implications of the feasibility findings for the design of the main study?
  This study demonstrates that combining three different components into one treatment is possible, and that it is possible to implement such an integrated treatment to this, often overlooked and under-resourced segment of the population. Second, findings from this study inform recommendations and ‘lessons learned’ for future applications and improvements of this integrated treatment.

## Background

The current number of older adults ages 65 years and older in the USA is about 54 million (i.e., 16% of the total) [1]. As the older population grows, challenges related to healthy aging will also intensify, and may diminish the likelihood that one can continue living in one’s own homes and communities. Unfortunately, many older adults experience physical frailty, declining health, and/or are financially ill equipped to address the physical, psychological, and environmental factors necessary for independent living [2]. In 2019, 4.9 million (8.9%) older U.S. adults were living below the poverty threshold, with a disproportionately high representation of Hispanic and Black older adults [3, 4]. Healthcare costs or those from an unexpected illness, and physical and mental health deterioration can result in a downward spiral in independent functioning, eventually leading to unwanted institutionalization [5]. “Aging in Place” is an increasingly popular concept that reflects the overwhelming preference of older adults to live out their lives in their own homes as opposed to a structured, typically healthcare focused (and almost universally more expensive) institutionalized setting [6]. This along with the potential social and economic burden of expanding institutionalized care for millions of older individuals is driving research and community efforts that will enable older people to remain in their homes rather than transitioning to care facilities. Successful initiatives are likely to include strategies that address environmental and situational factors that allow older adults to maintain their autonomy while sustaining, or even improving, the quality of their social connections [7].

Age-related physical challenges such as chronic pain, fatigue, and concomitant depression (which is often amplified by pain and fatigue) are quite common in older individuals and are considered significant barriers to ‘aging in place’. About 28% of older adults suffer from chronic pain [8] while ~ 31% report experiencing significant fatigue [9]. Chronic pain and fatigue can result in reduced physical activity, leading to decreased muscle tone and increased risk of injury from accidental falls; intensified arthritis-related impairment; and other conditions that reduce capacity to participate in activities of daily living or in recreational activities that sustain mental and physical well-being [10–13]. Interventions to address pain and fatigue can be effective with older adults, particularly those targeting mobility and psychosocial [14, 15]. However, these programs are not always available or accessible to older adults. This is especially true for low-income older adults due to logistic, financial, and geographic barriers [16, 17]. Moreover, many older adults may require more than simple strengthening programs to maintain healthy aging, for instance specific strategies or integrated treatments to improve mental health and engagement in addition to physical health. Very few health-mental health integrated ‘aging in place’ programs exist in the U.S. and even fewer have been evaluated for their impact on physical and psychological outcomes.

Home-based telemedicine technology may increase access to care and the ability of older adults to ‘age in place’. Unfortunately there is a paucity of literature regarding the use of mHealth (e.g., tablet based applications) and eHealth (e.g., televideo via tablet device) as a platform for managing pain in older adults [18, 19]. However, technology-based interventions to improve health outcomes in older adults are emerging and seem to improve engagement with providers while addressing barriers to care, with the same effectiveness as traditional settings (in office visits) [20–22]. To advance this important area of research, this pilot study evaluated the feasibility (as determined by measures related to recruitment, enrollment, retention, and satisfaction with treatment) of combining mindfulness (Gentle Yoga and Yogic Breathing, or “GYYB”) [23] and mental health components (Behavioral Activation for Depression, or “BA”) [24] with an existing, evidence-based muscle strengthening and balance retraining program, Otago (“OG”), endorsed by the Centers for Disease Control as an effective fall intervention program [25]. The overarching goal of the multicomponent OG + GYYB + BA intervention, *Activate for Life*, is to reduce pain and fatigue in lower-income older adults, ultimately improving overall physical functioning and mental health and increasing the likelihood that older persons are able to successfully ‘age in place’. For this report on feasibility, we hypothesized that the team would be able to: enroll 30 participants (10 participants per group) during the 18-month recruitment phase (not counting COVID-related study recruitment pause); deliver the integrated eHealth treatment via televideo; collect saliva samples for inflammatory biomarker analysis, and achieve high patient rated satisfaction (above 80%) and low treatment attrition (below 25%).

## Methods

### Participants

Older adults ages 60 years and older living in subsidized housing facilities or their own homes in the US Southeast were recruited via flyers placed in housing facilities or through direct referral by providers familiar with the project. Subsidized facilities included those maintained by non-profit organizations such as the Humanities Foundation and Housing and Urban Development programs located in urban and suburban communities in South Carolina. Interested participants initiated contact or requested that more information be presented by the study team at their facility. Enrollment was offered for those meeting the following inclusion criteria: English-speaking, any gender, aged 60 years or older, residing in low-income housing or meeting the definition of low income (defined as ≤ 150% of the official poverty threshold), and experiencing a pain score of ≥ 8 on the PROMIS Pain Interference short form. Excluded from participation in the study were those who had significant cognitive impairment or dementia (a score between 0 and 2 as measured by the Mini-Cog [26]; those unable or unwilling to give consent; those with a physical disability resulting in an inability to ambulate 150 feet (ft.) with or without the assistance of another individual or assistive device; and those who were unable/did not want to operate the provided tablet device. All screening questionnaires were administered following informed consent.

### Randomization

*F*or this 12-week pilot trial, we employed a randomized trial design and 3 × 3 repeated measures (treatment × time) approach to compare feasibility measures among participant groups in Arm 1 (OG), Arm 2 (OG + GYYB), and Arm 3 (OG + GYYB + BA; together, these comprise the full “AFL” intervention). After signing the informed consent document, 30 participants were randomly allocated to one of the 3 arms (*n* = 10 each) using a randomization scheme developed by the study biostatistician under the Research Electronic Data Capture (REDCap) system. Study data were obtained at baseline, post-treatment (12 weeks), and at 3-month follow-up. In addition to feasibility findings reported here, data collection also saliva-based monitoring of cortisol and inflammation levels and self-report of mood, pain, fatigue, physical balance, collected, as well as blood pressure and heart rate. These later clinical outcomes will be reported in a separate manuscript.

A computer-generated randomization strategy designed by the statistician (J.B.) was used by the study coordinator to assign enrolled patients to the three arms. This trial was not powered to test clinical outcome hypotheses, rather, these analyses were considered hypothesis generating and descriptive. The principal investigator (T.K.) was blinded to study assignment; the study coordinator who collected and entered data and participants were not. This study design was approved on June 5, 2018 by the Medical University of South Carolina (MUSC) Institutional Review Board (approval #Pro00076835) and registered at ClinicalTrials.gov (identifier NCT03853148) released February 22, 2019. Recruitment and enrollment commenced March 2019 and the study was completed August 2021 when the final participant finished the final 3-month follow-up. The study ceased recruitment when 30 participants were enrolled.

### Procedures

#### Feasibility outcomes and criteria for success

We focused feasibility on four main areas: recruitment, enrollment, retention, and patient satisfaction with treatment. For recruitment, we measured the number of completed treatment sessions completed by participants after treatment initiation. The expected number of sessions to be completed was 12 over the 12 weeks of treatment. We also measured the number of participants who successfully completed the 12-week treatment. The expected number of completers was about 70% of the participants. This percentage is based on evidence of normal dropout from clinical research in the integrated components [27–29].

### Components of treatment

#### Otago

OG is an evidence-based muscle strengthening and balance retraining program endorsed by the Centers for Disease Control as an effective fall intervention program [30]. The OG program (Arms 1, 2, and 3) encompasses a series of 17 warm-up exercises followed by additional specific exercises such as walking heal to toe, backwards, in a figure eight pattern, and side stepping to improve strength and balance.

#### Gentle yoga and yogic breathing

The GYYB component (Arms 2 and 3) was developed by study team member (SB) [31] who is an International Association of Yoga Therapists certified yoga instructor and GYYB was designed to improve overall flexibility, bodily control, and mindfulness in movements for older persons with limited mobility based on principles of integral yoga [30]. The study team previously developed a one-hour GYYB video reviewing: 1) Yoga postures followed by 2) Yogic breathing exercises.

### Behavioral activation

BA (Arm 3 only; the “full” AFL intervention) incorporates structured strategies for increasing patients’ engagement in values-based, social, and healthy activities, such as interacting with supportive family and friends, that are likely to produce reinforcement in the natural environment [32]. Daily planners and worksheets are used in conjunction with talk therapy to identify, plan, and rate behaviors that are easily incorporated into daily activities.

### Data collection and analysis

Feasibility study data were collected and managed using Research Electronic Data Capture (REDCap) tools. After consulting the literature on behavioral health feasibility studies and prior feasibility trials, and noting the “rule of thumb” for pilot studies (recommended sample size of 30), a sample size of 10 per condition, or 30 total was selected [33, 34]. This number also assured sufficient patient variability in each group to inform feasibility conclusions.

The analytic plan followed the standard approach wherein descriptive methods were used to characterize the sample, including percentage distributions, means, and standard deviations. For dropouts, the average session number at which participants decided to withdraw from treatment was noted. A Patient Satisfaction with Treatment survey was developed specifically for this study, with yes/no questions and 5-point Likert scale ranging from not at all satisfied to very satisfied, with subsequent opportunities to clarify their yes/no responses. Questions specifically targeted participants’ experiences with each component of the assigned Arm, including their impressions of the tablet devices, software, blood pressure monitoring, saliva sample collection (at baseline, mid treatment and end of treatment) and activity tracking. Data were analyzed using IBM SPSS Version 26.0. For open-ended questions, participant responses related to their experience with treatment were categorized by the contents of treatment and devices used.

## Results

### Demographics

Fifty-nine older adults ages 60 and older were approached and screened for this pilot study. Thirty met the inclusion criteria and agreed to be randomized in equal proportions to the three conditions and nineteen participants completed treatment. Table 1 presents demographic information for the 30 participants who were randomized to the three study arms. The average age of the full sample was 70.6 years (SD = 6.4 years). The majority of the participants were female, Black, and retired with some college-level education. Most reported taking anti-hypertensive, cholesterol, and/or pain medications. None of the participating older adults had experience with any of the three treatment components.

**Table 1:** Participant demographics and other characteristics

	Arm 1		Arm 2		Arm 3		Total	
Variable	*M*	SD	*M*	SD	*M*	SD	*M*	SD
Age (years)	73.4	5.6	68.5	7.5	69.8	5.4	70.6	6.4
Variable	n	%	n	%	n	%		
Sex								
Female	7	70%	7	70%	7	70%	21	70%
Race								
White	5	50%	5	50%	2	20%	12	40%
African American	5	50%	5	50%	8	80%	18	60%
Education								
Some college or more	6	60%	6	60%	5	50%	17	57%
Employment^a^								
Retired	8	80%	6	60%	5	50%	19	63%
Medication use								
Antihypertensive	5	50%	8	80%	5	50%	18	60%
Cholesterol	6	60%	5	50%	8	80%	19	63%
Pain	7	70%	4	40%	3	30%	14	47%

^a^ Employment category

### Feasibility

**Fig. 1:**
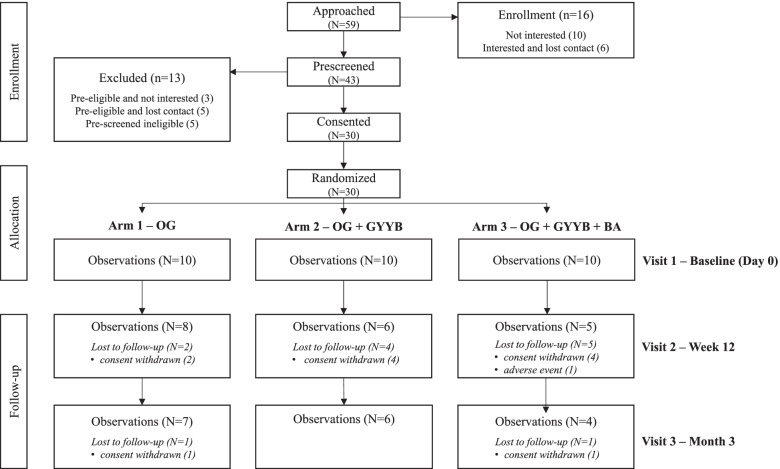
Consort Diagram

To recruit and enroll participants in this study, we used IRB approved flyers and presentations, as well as word of mouth (e.g., presentations at local senior centers). These strategies were implemented by study personnel at sites where low-income older adults frequent or live. Figure 1 shows the Consort diagram for this study. We approached 59 potentially eligible older adults, of which 10 were not interested and were interested but were unable to be recontacted for prescreening. We prescreened the remaining 43, 5 of whom were ineligible. Of the remaining eligible candidates 3 decided they were not interested and 5 were unable to be recontacted. The consenting and enrollment processes were initially conducted in person by study personnel at patient residence or recreational sites, and then moved to an online platform (first contact via telephone, then televideo calls were established to complete consent and baseline processes) due to pandemic restrictions. Both approaches were found to be feasible and acceptable by study participants.

The lowest rate of treatment attrition was observed for Arm 1, with 70% of the participants completing treatment. While overall retention was lowest for Arm 3, it is important to note that 80% of the participants were retained past session 7, with 50% completing the full treatment (session 12), despite the fact that involvement in the full AFL intervention required far more effort on the part of the participant. Most Arm 3 participants were able to complete about half the treatment sessions before dropping out, in contrast to dropouts from Arms 1 and 2 who exited the study shortly after completing baseline due to multiple schedule conflicts. Additionally, participants in Arm 3 encountered several unique circumstances that participants in the other two arms did not experience. Unfortunately, one patient died during treatment (after completing visit 7, due to a pre-existing condition) and another participant dropped out after visit 7 due to ongoing issues with technology (poor network connectivity in their location,). Finally, three participants dropped out due to schedule conflicts—two at visit 6 and one at visit 11. Table 2 provides information about the number of completed treatment sessions for each arm.

**Table 2:** Quantitative feasibility outcomes regarding treatment completion

Treatment^a^ condition	% < 2 visits	% 3–6 visits	% 7–12 visits	% with follow-up	Reason for dropout
Arm 1	20%	0%	80%	70%	Conflict of schedule
Arm 2	40%	0%	60%	60%	Conflict of schedule, pain
Arm 3	0%	20%	80%	40%	Death, pain, device(s) issues

^a^ *Arm 1* Otago, *Arm 2* Otago +GYYB, *Arm 3* Activate for Life

### Satisfaction

Satisfaction questions included specific queries about the characteristics and components of the intervention and associated technologies, such as using electronic devices (i.e., activity tracker, blood pressure device, tablet). as well as open questions regarding those components. After completion of study, 19 participants were available to respond to the questions regarding satisfaction. Overall, 85% of Arm 1 participants reported high satisfaction with the physical exercise program as compared to 67% in Arms 2 and 3 (when combining % vs total N of both groups). Specifically, 71.4% of the participants in Arm 3 reported positive experience with the treatment and interaction with the research team, nonetheless, some participants commented that it did require effort to organize their time so that they could incorporate all of the activities. Another relevant comment was regarding the age difference between the therapist and the clients, insofar as some clients thought that the 50-year age difference between the therapist and patients was too large. Participants who dropped out from treatment reported they were unable to continue for personal reasons rather because of any characteristics of the integrated treatment. Finally, participants in Arms 2 and 3 reported lower satisfaction with the GYYB component in their open responses. From our interview findings, participants opted not to answer the question regarding GYYB experience (How would you rate overall satisfaction with the GYYB, from satisfied to somewhat dissatisfied). There was an overall lack of engagement with GYYB and patients had difficulty remembering the GYYB exercises when they were asked about their experience with them, likely because they were not performing the activity daily.

The majority of the older adults in this study reported a low level of experience operating a tablet device. For example, 85% of Arm 1 participants and 60% of participants in both Arm 2 and Arm 3 indicated they did not have experience with an iPad/tablet. Learning how to use the iPad/tablet (ease of use) was largely reported to be easy, with 57% of Arm 1 participants rating the tablet as “easy” or “somewhat easy” to use and 42% “somewhat difficult” (in Arm 2, 50% reported that the tablet was “easy” to use versus “somewhat difficult,” while in Arm 3 83% of participants rated the tablet as easy). The majority of the participants in this study (57% of Arm 1, 83% of Arm 2, and 33% of Arm 3) encountered at least one issue while using the iPad/tablet. Most issues were related to connectivity or resetting the tablet device so that the activity tracker could upload/sync data correctly. Some reported that once they were able to sort out the issues they were “ok” with the device, whereas others felt that it was too much trouble and was part of the reason for dropout. Finally, most participants found the blood pressure monitor “easy” to use (100% in Arm 1 and 83% in both Arms 2 and 3) while responses regarding issues encountered using either the blood pressure monitor or the activity tracker (29% in Arm 1, 67% in Arm 2, and 50% in Arm 3) varied across arms. Most issues were related to the device battery life; not understanding the screen of the blood pressure device; skin irritability from the activity tracker, requiring participants to switch from one arm to the other; discomfort; and problems syncing with app (the most commonly reported issue).

### Continuing treatment

The majority of the participants indicated they continued performing the OG physical activities they learned during the intervention after completion of treatment (71% in Arm 1, 67% in Arm 2, and 75% in Arm 3). Participants in all three arms reported exercising an average of twice a week. None of the participants in Arms 2 and 3 continued performing GYYB exercises.

Finally, the research team asked open-ended questions of all participants to describe what they thought were the best and worst parts of the program. Positive aspects included learning about their own ability to challenge themselves, learning how to use the iPad tablet device, and ability to self-monitor their own blood pressure. They also expressed a generally positive attitude about being required to log in to the tablet every day to complete tracking activities and blood pressure measurement. They reported that they were motivated and encouraged to complete the intervention because other participants or residents from the same community were involved in the study and this was another way to build friendships. About 40% of participants reported that initially they did not want to exercise but that once they started participating they wanted to continue. In Arms 2 and 3 there appeared to be a lack of engagement with the GYYB exercises by 60% of the participants. Verbal reports of participants indicated difficulty understanding the purpose of the GYYB exercises and feeling that they would have liked to see videos that were more targeted to them (for instance, another older adult demonstrating the exercises). Participants also indicated they did not like the writing activities that were part of BA. Specifically, some participants disliked writing down daily activities because they did not want other people (including the therapist or anyone on the study team) to know what they were doing. Also, some had stopped writing due to complications from arthritis and being asked to do this activity was physically challenging. Some participants indicated that it was challenging to complete exercises, particularly when pain was present, or that it was difficult to find motivation to do the exercises on their own. Wearing the activity tracker was uncomfortable for some, and difficulties syncing with the app due to connectivity issues introduced additional challenges. Finally, we collected saliva to determine changes of cortisol and other inflammatory levels and patients did not have report any difficulties with data collection procedures. However, at least four reported having difficulty producing the amount of saliva required due to dry mouth, but despite this, all participants had no problems with being requested to do this part of the study.

## Discussion

Older adults living in low-income communities face tremendous economic and health-related challenges that diminish their ability to ‘age in place’ [34]. Insufficient access to care, particularly in rural areas, exacerbates these challenges and their effects on health. Advances in technology may help to overcome these barriers through increased access to integrated mHealth and physical health interventions focused on improving mood, mobility, and overall well-being [35] that negatively affect ‘aging in place’. The present pilot study examined the feasibility of introducing varied levels of integration for existing interventions addressing physical [36], functional [37], and psychological [38] factors that might help independently residing, albeit resource-poor older adults, successfully age in place. We hypothesized that this integrated treatment would not only satisfy our feasibility criteria related to recruitment, enrollment, and retention, but would also be viewed as particularly welcome when delivered via telemedicine, with patients reporting high satisfaction with the program. Our results support the feasibility of the mHealth-delivered intervention and its component parts, insofar as lower income older adults agreed to be randomized to condition, accept conditions of treatment components, and complete 6 or more sessions. Satisfaction with the mHealth component of the *Activate for Life* intervention was also high.

Overall, about three quarters of study participants reported high satisfaction with the OG physical exercise components of treatment, while participants reporting severe pain were more likely to drop out of the study. Experience with the iPad varied widely within the arms of this study. The main complaints were not related to the technology per se, but rather how the technology was performing due to connectivity issues such as poor Wi-Fi signal for some of the participants. This prevented a few participants from full participation in the televideo sessions or made operating the mHealth app more cumbersome (i.e., the activity tracker would not synchronize with the app or videos would run slower or with pauses). Indeed, participants did enjoy using the blood pressure monitor and were more tolerant when experiencing technical issues with this device because they clearly understood that the information it provided was important (note, the majority of these patients were taking antihypertensive medications). This underscores the importance of clearly explaining why certain devices and exercises are being used in the study to participants, since insufficient information combined with technical difficulties resulted in frustration.

Impressively, many of those who completed treatment reported that they continued to perform the exercises they had learned after they finished the study, with at least twice a week engagement in those activities. Exercise routines for older adults may require more than 12 weeks of training, particularly if the exercises are new [39, 40]. Some evidence indicates that older adults feel more comfortable doing things they enjoy and have already experienced in the past [41], so interventions that modify existing exercise patterns are likely advisable for increasing adherence to physical regimens in this patient population. Another positive observation was the enthusiasm reported by the older adults who learned that other people in their community were participating in the study. Social support and social connection are highly important for all age groups but may be particularly so for older adults engaging in novel activities to sustain motivation [42]. Because most activities were conducted individually and within the home, we might have increased satisfaction and retention if we had included opportunities to engage in treatment components with other participants, perhaps virtually and in group settings.

## Study limitations/identified issues of treatment implementation

Limitations of this study include its small sample size which limits generalizability of the results, limited representation of participants from minority and rural populations, limited follow-up periods post intervention, and issues with technology in terms of internet connectivity. We encountered problems that were related to the community-based nature of our implementation, including issues of internet connectivity. This issue seemed to drive dropout, despite the fact that the study team tried to provide solutions for connectivity issues. Because the Otago component of the treatment is more commonly used in residential settings [35], it will be important to anticipate and prevent these problems in future studies with lower-income older adults to avoid negative experiences or discontinuation.

We also perceived that our participants may not have been able to form fully effective therapeutic relationships with our young therapists in Arm 3 to the extent that an older provider might have. Indeed, participants expressed this to our therapists and noted the large gap in age (of about 50 years). The preference of older adults with respect to matching with a provider similar to themselves appeared to extend beyond age, to also include preferences for matching in terms of appearance, gender and culture. Specifically, a recurring theme our team noted was participant lack of engagement with the GYYB component of treatment and with Behavioral Activation therapists. As this was a small, localized sample with limited representation of older adults, we believe that engagement may vary depending on the region of the country. Future considerations should include assurances that exercises are presented considering age, gender, and cultural background similar to the specific target sample, to increase engagement with the activity.

The COVID-19 pandemic and subsequent public health advisory restrictions made it particularly difficult for older adults to engage with study personnel. The justifiable concern of older adults regarding close contact with study personnel such as during visits for resolving technical issues, or saliva sample collection was a major issue. Nonetheless, both the research team and participants showed extraordinary motivation to complete measures and to overcome these obstacles/restrictions, particularly concerning saliva sample collection (for example, use of additional protective gear). We believe this indicates that, under normal conditions, far fewer difficulties would be encountered. Unrelated to this, another limitation was underrepresentation of minority groups. Better representation would have been preferable.

### Implications for physical, functional, and behavioral health

Future studies of sufficient sample size should address lessons learned in this feasibility study, particularly with respect to attention to cultural/geographical issues, broadband connectivity issues, and efforts to increase social interaction during treatment components. Consideration of cultural and age representation, and appropriate language, for instance, using simpler wording to refer to activities for breathing or to support mood in video and print/tablet applications may increase engagement, participation, and adherence to treatment. Peers may also be a useful addition to treatment components and can help to address many of the aforementioned issues, such as resolving technical issues (to avoid frustration), personal contact to motivate oneself to complete the activities, or seeing/interacting with people they know in their community who successfully have completed treatment or know how to do the most challenging activities such as the GYYB or following the planned activities of Behavioral Activation. Finally, as pain was reportedly related to dropout in this small sample, future studies should consider implementing integrated strategies to address pain in combination with components of the Activate for Life treatment to increase retention. As COVID-19 was an unexpected event that challenged participants, research teams, and housing agencies, high levels of innovation and flexibility were brought to bear, and these modifications, along with mHealth and telehealth may help address the challenges noted in delivering this type of multidimensional treatment to older adults.

## Supplementary Information

**Additional file 1.** End of Study Survey.

## Data Availability

Not applicable.
