# Supplementary material for: Feasibility trial of an integrated treatment “Activate for Life” for physical and mental well-being in older adults
Source: Pilot Feasibility Stud. 2022 Feb 11;8:38. doi: 10.1186/s40814-022-01000-8 (PMC8832080; doi:10.1186/s40814-022-01000-8)
Supplement: Supplementary file 1 — Additional file 1. End of Study Survey. [file 40814_2022_1000_MOESM1_ESM.pdf]

A mHealth intervention for improving physical, functional, and physiological outcomes in community-dwelling older adults: A randomized controlled pilot trial

End of Study Exit Survey

<u>All arms</u>
How would you rate your overall satisfaction with the physical exercise program?

Satisfied
Somewhat satisfied
Neutral
Somewhat dissatisfied
Dissatisfied

<u>Otago + Yoga & Ottago + Yoga + BA arms only</u>
How would you rate your overall satisfaction with the Yoga breathing relaxation program?

Satisfied
Somewhat satisfied
Neutral
Somewhat dissatisfied
Dissatisfied

<u>Ottago + Yoga + BA arms only</u>
How would you rate your overall satisfaction with having a Coach to help you reach your exercise goals?

Satisfied
Somewhat satisfied
Neutral
Somewhat dissatisfied
Dissatisfied

<u>All arms</u>
Before you joined the study, how much experience would you say you had with an iPad tablet?

None at all
Very little
A little
Some
A lot

Did you find learning how to use the iPad tablet ‘easy’, ‘somewhat easy’, ‘somewhat difficult’, or ‘difficult’?

Easy
Somewhat easy
Somewhat difficult
Difficult

Did you encounter any problems while using the tablet?

Yes
No

If ‘Yes’, what problems did you have? _____

Did you find using the cuff and blood pressure monitor ‘easy’, ‘somewhat easy’, ‘somewhat difficult’, or ‘difficult’?

Easy
Somewhat easy
Somewhat difficult
Difficult

Did you encounter any problems with the cuff and/or blood pressure monitor?

Yes
No

If ‘Yes’, what problems did you have? ______

Did you find using the daily activity tracker ‘easy’, ‘somewhat easy’, ‘somewhat difficult’, or ‘difficult’?

Easy
Somewhat easy
Somewhat difficult
Difficult

Did you encounter any problems with activity tracker while you were in the study?

Yes
No

If ‘Yes’, what problems did you have? ______

During the follow-up 12 weeks after you finished the study, did you continue doing any physical activity exercises you learned?

Yes
No

If ‘Yes’, how many days a week would you say you did them? (1-7 days)

1
2
3
4
5

6
7

Do you still do these exercises now?

Yes
No

<u>Ottago + Yoga + BA arms only</u>
Did you find using the tablet to do your video coaching session 'easy', 'somewhat easy', 'somewhat difficult', or 'difficult'?

Easy
Somewhat easy
Somewhat difficult
Difficult

Did you encounter any problems with the tablet while doing your coaching sessions? (Y/N)

Yes
No

If 'Yes', what problems did you have? ______

<u>Otago + Yoga & Ottago + Yoga + BA arms only</u>

During the follow-up 12 weeks after you finished the study, did you continue doing the yoga breathing exercises you learned?

Yes
No

If 'Yes', how many days a week would you say you did them? (1-7 days)

1
2
3
4
5
6
7

Do you still do yoga breathing now?

Yes
No

If 'Yes', how many days a week would you say you did them? (1-7 days)

1

2
3
4
5
6
7

<u>All arms</u>

What was the best part of being in the study?

What was the worst part of being in the study?

Would you volunteer again for a research study?

Yes
No

If "'Yes', can we call you if we have a study that we think you might be eligible for?

Yes
No

Do you have any other comments that you like to make about the study?
